# The cAMP Pathway Amplifies Early MyD88-Dependent and Type I Interferon-Independent LPS-Induced Interleukin-10 Expression in Mouse Macrophages

**DOI:** 10.1155/2019/3451461

**Published:** 2019-04-17

**Authors:** Orna Ernst, Yifat Glucksam-Galnoy, Muhammad Athamna, Iris Ben-Dror, Hadar Ben-Arosh, Galit Levy-Rimler, Iain D. C. Fraser, Tsaffrir Zor

**Affiliations:** ^1^Department of Biochemistry & Molecular Biology, School of Neurobiology, Biochemistry & Biophysics, Life Sciences Faculty, Tel Aviv University, Tel Aviv 69978, Israel; ^2^Signaling Systems Section, Laboratory of Immune System Biology, National Institute of Allergy and Infectious Diseases, National Institutes of Health, Bethesda, 20892 MD, USA; ^3^Triangle Regional Research and Development Center, P.O. Box 2167, Kfar Qari 30075, Israel

## Abstract

Interleukin-10 (IL-10) is a key anti-inflammatory cytokine, secreted by macrophages and other immune cells to attenuate inflammation. Autocrine type I interferons (IFNs) largely mediate the delayed expression of IL-10 by LPS-stimulated macrophages. We have previously shown that IL-10 is synergistically expressed in macrophages following a costimulus of a TLR agonist and cAMP. We now show that the cAMP pathway directly upregulates IL-10 transcription and plays an important permissive and synergistic role in early, but not late, LPS-stimulated IL-10 mRNA and protein expression in mouse macrophages and in a mouse septic shock model. Our results suggest that the loss of synergism is not due to desensitization of the cAMP inducing signal, and it is not mediated by a positive crosstalk between the cAMP and type I IFN pathways. First, cAMP elevation in LPS-treated cells decreased the secretion of type I IFN. Second, autocrine/paracrine type I IFNs induce IL-10 promoter reporter activity only additively, but not synergistically, with the cAMP pathway. IL-10 promoter reporter activity was synergistically induced by cAMP elevation in macrophages stimulated by an agonist of either TLR4, TLR2/6, or TLR7, receptors which signal via MyD88, but not by an agonist of TLR3 which signals independently of MyD88. Moreover, MyD88 knockout largely reduced the synergistic IL-10 expression, indicating that MyD88 is required for the synergism displayed by LPS with cAMP. This report delineates the temporal regulation of early cAMP-accelerated vs. late type I IFN-dependent IL-10 transcription in LPS-stimulated murine macrophages that can limit inflammation at its onset.

## 1. Introduction

Stimulation of macrophages with the TLR4 ligand, LPS, results in the production of cytokines, chemokines, and reactive oxygen and nitrogen species which drive inflammation [1, 2]. Yet LPS also stimulates the expression of the anti-inflammatory cytokine IL-10 with a time lag that results from the requirement for autocrine activity of type I interferons (IFNs) [3–8]. The combination of a TLR ligand and a second signal, such as an IgG immune complex, apoptotic cell remnants, or a cAMP inducer, generates a different population of macrophages that limit inflammation by enhanced IL-10 secretion and reduced expression of proinflammatory cytokines [2]. These IL-10-producing cells have an important regulatory role in attenuating the development of chronic autoimmune pathological states as well as acute endotoxemia [9]. Consistently, polymorphisms in the IL-10 gene region are associated with autoimmune pathologies, including ulcerative colitis, type I diabetes, severe juvenile rheumatoid arthritis, and Behcet's disease [10]. Furthermore, mutations in the IL-10 receptor are associated with inflammatory bowel disease (IBD) in human [10], while IL-10 knockout mice readily develop IBD [11]. Considering the crucial role that IL-10 plays in restoring homeostasis and preventing damage to the host and inflammatory autoimmune pathologies, a comprehensive understanding of the mechanisms regulating IL-10 expression is essential.

We have previously shown that costimulation of macrophages with LPS and cAMP inducers for a short period of 2 h results in synergistic IL-10 transcription, while either stimulus alone is largely ineffective [12]. Furthermore, a cell-permeable cAMP analog, but not a selective agonist of exchange protein directly activated by cAMP (EPAC), can synergistically elevate LPS-stimulated IL-10 secretion [13], while inhibition of PKA completely abolishes the amplification of IL-10 expression by the cAMP inducer isoproterenol (*β*-adrenergic receptor (*β*-AR) agonist), but does not significantly affect the low IL-10 expression in cells stimulated by LPS alone [14]. To demonstrate maximal synergism between cAMP and LPS, these previous studies examined the early phase of IL-10 expression (2-4 h), in which LPS alone has a minimal effect. How cAMP elevation affects LPS-stimulated IL-10 secretion in longer incubations is largely unknown. It is in particular intriguing as delayed expression of the anti-inflammatory IL-10 assures a proper inflammatory response to infection. Indeed, significant expression and secretion of type I IFN (i.e., IFN*α* and IFN*β*) from macrophages are achieved within several hours of LPS stimulation, and their autocrine activity is required for quantitative IL-10 expression [3–8]. A neutralizing antibody against the common type I IFN receptor subunit, IFN*α*R1, was unable to block early phase (3 h) IL-10 secretion from macrophages stimulated by LPS alone or together with CGRP, a cAMP-elevating neuropeptide that elevated IL-10 secretion by twofold [3].

The objective of the present research was to determine the timecourse of synergism between LPS and cAMP inducers and to examine the role of type I IFN in IL-10 expression in costimulated macrophages. We found that cAMP elevation can amplify only early, but not late, LPS-stimulated MyD88-mediated IL-10 expression. Unlike early LPS activity, autocrine/paracrine type I IFN activity which accounts for IL-10 induction at the late stage does not synergize with the cAMP pathway. The synergism between cAMP-elevating agents and early type I IFN-independent LPS signaling enables efficient and accelerated IL-10 transcription that can limit inflammation at its onset in specific contexts.

## 2. Materials and Methods

### 2.1. Reagents

Lipopolysaccharide (LPS; *Escherichia coli* serotype 055:B5), isoproterenol, imiquimod, and polymyxin B were purchased from Sigma-Aldrich (St. Louis, MO). XTT, L-glutamine, FBS, and penicillin-streptomycin-nystatin were purchased from Biological Industries (Beit Haemek, Israel). DMEM, OptiMEM, were purchased from Gibco. Rolipram was purchased from Spectrum Chemical (New Brunswick, NJ). Poly(I:C) was purchased from Invivogen (Toulouse, France). Pam_2_Cys-SKKKK (hereafter, Pam2Cys) was purchased from EMC Microcollections (Tuebingen, Germany). ELISA reagent sets for IL-10 and TNF*α* were purchased from R&D Systems (Minneapolis, MN). The mouse IL-10 promoter luciferase reporter gene construct, a kind gift from Dr. S. Smale [15], was amplified using DH10B bacteria (Invitrogen, Carlsbad, CA) and purified using an Endofree Plasmid Maxi Kit (Qiagen, Hamburg, Germany). HD-fugene and TransIT2020 transfection reagents were purchased from Roche (Mannheim, Germany) and Mirus Bio (Madison, WI), respectively. A dual-luciferase reporter assay kit was from Promega (Fitchburg, WI). Mouse IFN*α* was from Miltenyi Biotec (Bergisch Gladbach, Germany). The MasterPure RNA purification kit was from Epicentre Biotechnologies (Madison, WI), the High Capacity cDNA Reverse Transcription kit, and the SYBR green reagent were purchased from Applied Biosystems (Foster City, CA). PCERA-1 (phospho-ceramide analogue-1; chemical name: 1-methyl-2-(3-methoxyphenyl)-2-(octanoylamino)ethyl-disodium-phosphate) was synthesized as previously described [16, 17] and kindly supplied by Dr. Nathanael Gray.

### 2.2. Animal Care

Male BALB/c WT mice and female C57BL/6 WT and MyD88 knockout mice, obtained from the animal breeding center of Tel-Aviv University (TAU), were housed in a pathogen-free room under controlled temperature (22-23°C), humidity, and lighting (12 hours light-dark cycles) and were given access to food and water ad libitum. Animal care and experimentation were carried out in accordance with TAU guidelines (Approval no. L-05-007). CO_2_ was used to sacrifice the mice.

### 2.3. Cell Culture

Mouse RAW264.7 macrophage cells were obtained from American Type Culture Collection (ATCC, Rockville, MD). The cells were grown to 80-90% confluence in DMEM medium supplemented with 8 mM L-glutamine, 100 U/ml penicillin, 100 *μ*g/ml streptomycin, and 1250 U/ml nystatin (hereafter, culture medium) and with 10% FBS, at 37°C in a humidified incubator with 5% CO_2_.

### 2.4. Isolation of Bone Marrow-Derived Macrophages (BMDM)

Female C57BL/6 WT and MyD88 knockout mice (6-8 weeks) were sacrificed, and the femoral and tibial marrow was flushed with culture medium supplemented with 10% FBS using a 26-gage needle. Following centrifugation, the cells were resuspended in culture medium supplemented with 20% FBS and 30% L929 cell conditioned medium (M-CSF source), seeded in Petri dishes at a density of 5.6^∗^10^4^ cells/cm^2^, and incubated at 37°C in a humidified incubator with 5% CO_2_. After 2 days, fresh medium was added. On day 7, the culture medium was replaced and the adherent cells (differentiated BMDM, ~98% homogenous by appearance) were transferred to storage in liquid N_2_ until used.

### 2.5. In Vivo Cytokine Expression

Male BALB/c mice (8-13 weeks, 23 ± 2 grams) were IP injected (0.1 ml) with either PCERA-1 (1 mg/kg) or saline, 30 min before an IP injection (0.1 ml) of LPS (5 mg/kg). Blood was obtained by cardiac puncture at 0-5 h, and serum cytokine levels were determined by ELISA. The data were expressed as the mean ± SEM of 3-6 animals per group.

### 2.6. In Vitro Cytokine Expression

RAW264.7 macrophages and BMDM were maintained for 48 h and 24 h, respectively, prior to the experiment in 96-well plates, at 1.0·10^5^ cells per well, in culture medium supplemented with 5% FBS, up to a confluence of 90%. The culture medium was replaced 2 h before treatment in order to avoid the artifact of medium replacement on signaling [18]. The cells were stimulated with LPS (10 ng/ml) and/or isoproterenol (1 *μ*M) at 37°C for 3-24 h. IL-10 secretion to the medium was measured by ELISA, according to the manufacturer's instructions, using a microplate reader (BioTek, Winooski, Vermont). The samples were stored at -80°C until used.

### 2.7. Real-Time PCR

The mRNA levels of IL-10 and HPRT in RAW264.7 cells were quantified by real-time PCR. The cells were seeded in a 24-well culture plate at 1.5·10^5^ cells per well and cultured for 48 h in culture medium supplemented with 10% FBS. The cells were then treated with LPS (10 ng/ml) in the presence or absence of isoproterenol (1 *μ*M) at 37°C for 1-24 h. Total RNA was isolated using the MasterPure RNA purification kit, and 1 *μ*g of RNA from each sample was reverse transcribed into cDNA using the High Capacity cDNA Reverse Transcription kit. Quantification was performed with 5 ng cDNA on the ABI Prism one step Sequence Detection System (Applied Biosystems), using SYBR green. The forward and reverse primer sequences were as follows (respectively): IL-10—CAGGGATCTTAGCTAACGGAAA and GCTCAGTGAATAAATAGAATGGGAAC; HPRT—GCGTCGTGATTAGCGATGATGAAC and CCTCCCATCTCCTTCATGACATCT.

### 2.8. Transfection and Reporter Gene Assay

RAW264.7 macrophages were grown for 24 h in 12-well plates, at 3·10^5^ cells per well, in culture medium supplemented with 10% FBS. The cells were then transfected for 24 h with 0.6 *μ*g of reporter plasmid and 0.2 *μ*g of Herpes Simplex Virus TK-renilla luciferase (for normalization). The plasmids were initially incubated with HD-fugene or TransIT2020 transfection reagent in OptiMEM for 15 min at room temperature. Following transfection, the cells were washed and stimulated with LPS (10 ng/ml) and/or isoproterenol at 37°C for 3-24 h, after which luciferase activity in cell extracts was determined following the manufacturer's instructions. Data were expressed as a ratio of IL-10 promoter-driven luciferase activity divided by the Renilla luciferase activity. Transfection with the empty reporter vector (pGL2B or pTAL) yielded no detectable activity. The DNA transfection efficiency of the macrophages was estimated to be <5% based on the fluorescence microscopy analysis of GFP expression plasmid transfection.

### 2.9. Type I IFN Activity Assay

A luciferase reporter ISRE-L929 cell line was a kind gift from Dr. Anat Herskovits [19, 20]. The ISRE-L929 cells were maintained for 24 h prior to the experiment in 96-well plates, at 0.5·10^5^ cells per well, in culture medium supplemented with 10% FBS, up to a confluence of 90%. The cells were then incubated for 4 h with 30 *μ*l of conditioned media from treated macrophages in a total volume of 100 *μ*l. Luciferase activity was measured as above.

### 2.10. Construction of Plasmids

The CRE consensus x4 (GGGAGTGACGTCAATGGA) heterologous reporter construct was generated using double stranded presynthesized oligonucleotides (Hylabs, Israel) cloned into the pTAL vector (Clontech, CA). Sequence verification was performed using the ABI PRISM 3100 Genetic Analyzer sequencer. Plasmid production was done using an Endofree Plasmid Maxi Kit.

### 2.11. Statistical Analysis

Data were analyzed using Student's *t*-test wherever applicable. In all cases, differences of *p* < 0.05 were considered to be significant. The number of biological samples appears in the legends. All experiments were repeated at least twice.

## 3. Results

### 3.1. Upregulation of LPS-Induced IL-10 Expression by cAMP-Elevating Agents Occurs Only at the Early Phase

**Figure 1:**
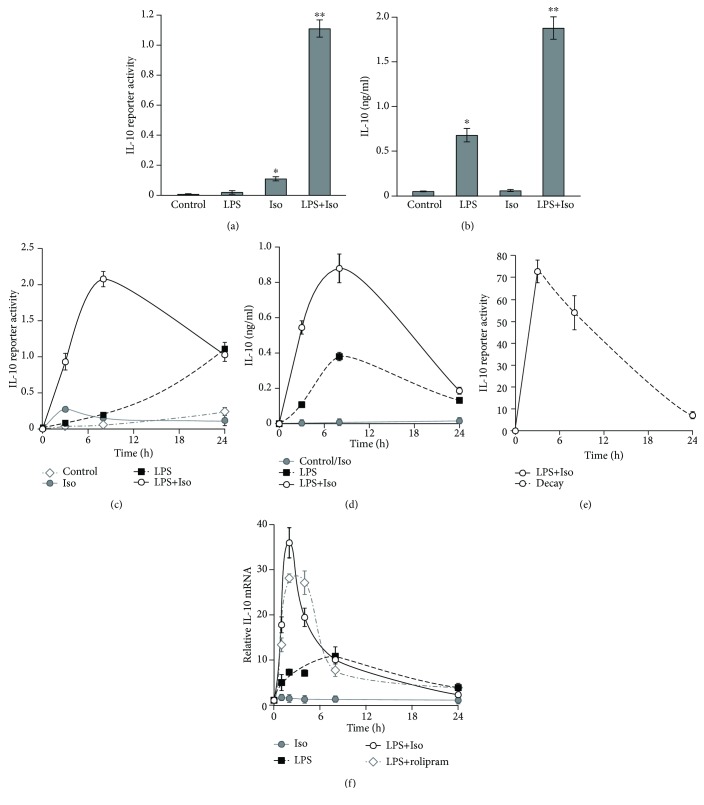
The cAMP pathway synergistically stimulates early, but not late, LPS-induced IL-10 transcription. (a–d) Early synergism at the IL-10 promoter reporter and IL-10 secretion levels. RAW264.7 macrophages, transfected with a full mouse IL-10 promoter reporter construct, were incubated with LPS (10 ng/ml) and/or isoproterenol (Iso, 1 *μ*M) for 3 h (a, b) or for up to 24 h (c, d). (b, d) The medium was collected, and the secretion of endogenous IL-10 was measured by ELISA. Data represent the mean ± SD (*n* = 3). (a, c) Luciferase reporter data expressed as the mean ± SD (*n* = 3) of values normalized against Renilla luciferase activity. (a, b) ^∗^ *p* < 0.003 and ^∗∗^ *p* < 0.0005 relative to resting cells. (c) *p* < 0.05 relative to resting cells for all treatments except for LPS at 3 h and Iso at 24 h. (d) Values of isoproterenol treatment were indistinguishable from control values. *p* < 0.05 relative to resting cells for LPS and for LPS+Iso at all time points. (c, d) *p* < 0.05 for LPS+Iso relative to LPS only at 3 h and 8 h. The experiments shown in (a–d) were carried out 45, 48, 14, and 12 times, respectively, with similar results. (e) Decay rate of the reporter. RAW264.7 macrophages, transfected with a full mouse IL-10 promoter reporter construct, were incubated with LPS (10 ng/ml) and isoproterenol (Iso, 1 *μ*M) for 3 h, the stimuli-containing medium was removed, and decay of the reporter was measured at the indicated time points along the dashed line. Luciferase reporter data expressed as the mean ± SD (*n* = 3) of values normalized against Renilla luciferase activity, relative to unstimulated control cells. *p* < 0.05 relative to resting cells at all time points. The experiment was carried out twice. (f) Early synergism at the IL-10 mRNA level. RAW264.7 macrophages were stimulated with LPS (10 ng/ml) and/or isoproterenol (Iso, 1 *μ*M) or the PDE4 inhibitor rolipram (20 *μ*M) for the indicated time. Total RNA was isolated from the cells, and IL-10 mRNA levels were assessed by real-time PCR. The intensity of IL-10 mRNA in unstimulated cells, normalized by HPRT mRNA, was set to 1. Data represent the mean ± SD (*n* = 3). *p* < 0.05 relative to resting cells for Iso at 1 h only and for all other treatments at all time points. *p* < 0.05 for LPS relative to LPS+Iso or LPS+rolipram only at 1-4 h. The experiment was carried out twice.

We have previously shown that various cAMP inducers, such as isoproterenol, PGE_2_, and the synthetic phospholipid PCERA-1, induce early IL-10 transcription in mouse macrophages in synergism with LPS [12]. The molecular mechanism and timecourse of the synergistic effect remained open questions. Therefore, we initially explored the time dependency of IL-10 expression in RAW264.7 macrophages stimulated by LPS in the presence or absence of the established cAMP inducer, isoproterenol. At 3 h, IL-10 promoter activity was modestly induced by isoproterenol and only marginally induced by LPS, whereas costimulation was nearly 10-fold stronger than the sum of separate activities (Figure 1(a)). Interestingly, LPS, but not isoproterenol, was able to stimulate endogenous IL-10 secretion at 3 h and costimulation was again synergistic (Figure 1(b)). These results suggest that cAMP plays a permissive and synergistic role in IL-10 protein expression at the early phase, as follows: (1) isoproterenol stimulates IL-10 transcription, but is unable to upregulate a limiting posttranscriptional step. (2) In contrast, while LPS cannot significantly stimulate early phase transcription unless a cAMP-elevating agent is present, it independently upregulates a limiting posttranscriptional step. At later time points, 8 h and 24 h, isoproterenol alone had only a minor effect on IL-10 promoter reporter level and no effect on endogenous IL-10 level (Figures 1(c) and 1(d)). Therefore, in the following IL-10 secretion and IL-10 promoter reporter experiments with RAW264.7 macrophages, the effect of isoproterenol was examined only in the context of LPS-stimulated cells (rather than resting cells). The synergistic effect of isoproterenol on LPS-induced expression of both IL-10 promoter reporter (Figure 1(c)) and endogenous IL-10 (Figure 1(d)) was highest at 3 h (early phase), partially reduced at 8 h (mid phase), and abolished at 24 h (late phase). The observed reduction between 8 h and 24 h suggested that the reporter half-life is shorter than this time interval and that therefore the measurement represents, at least in part, a freshly expressed rather than accumulated reporter. Indeed, transient stimulation of IL-10 promoter reporter expression (3 h) and measurement of its decay over the following 21 h showed that 90% of the IL-10 promoter reporter was degraded in that time window (Figure 1(e)). Similarly, the reduction in secreted IL-10 can be explained by its degradation or alternatively by sequestration. The time-dependent effect of isoproterenol on LPS-stimulated IL-10 expression was recapitulated also at the mRNA level, at which the high synergism observed at 1-2 h was partially reduced at 4 h and completely abolished already at 8 h (Figure 1(f)). Notably, LPS alone largely elevated IL-10 mRNA at all time points, while isoproterenol alone slightly increased IL-10 mRNA in a statistically significant manner only at the earliest time point, 1 h (Figure 1(f)). Taken together, with the observed effects of these stimuli on IL-10 protein secretion and reporter activity (Figures 1(a) and 1(b)), our data suggest that at the early phase neither LPS alone nor isoproterenol alone efficiently stimulate IL-10 transcription, whereas their combination synergistically drives IL-10 transcription. Furthermore, at the early phase, there is a clear contrast between the large effect of LPS on IL-10 mRNA level and its minor effect on IL-10 promoter activity. As the mRNA level reflects both transcription and decay rates, whereas the reporter assay accounts only for IL-10 promoter activation (hence transcription), our data suggest that LPS alone moderately increases IL-10 mRNA stability and subsequent protein expression and secretion at the early phase. Finally, the consistency of these various assays regarding costimulation indicates that the direct regulation of IL-10 promoter reporter activity in LPS-stimulated cells by cAMP reflects IL-10 mRNA and protein expression regulation by this pathway in the most sensitive manner.

**Figure 2:**
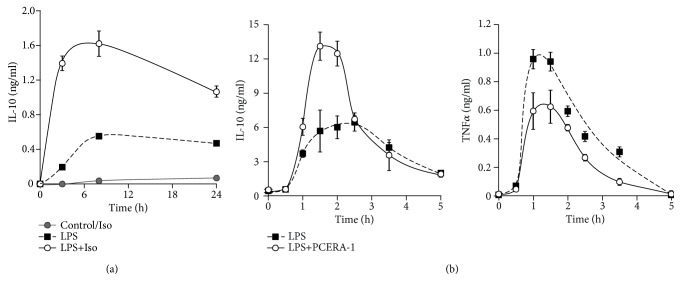
Early synergistic elevation of LPS-stimulated IL-10 expression by the cAMP pathway in BMDM and *in vivo* is diminished with time. (a) BMDM from C57BL/6 mice were incubated with LPS (10 ng/ml) and/or isoproterenol (Iso, 1 *μ*M) for up to 24 h. The medium was collected, and secretion of endogenous IL-10 was measured by ELISA. Data represent the mean ± SD (*n* = 6). Values of isoproterenol treatment were indistinguishable from control values. *p* < 0.05 for LPS+Iso relative to either LPS or control and for LPS relative to control, at all time points. (b) BALB/c mice were IP-injected with the cAMP inducer PCERA-1 (1 mg/kg) or with vehicle 30 min prior to IP administration of LPS (5 mg/kg). Blood was collected at the indicated time points. IL-10 and TNF*α* serum levels were measured by ELISA. Data expressed as the mean ± SEM (*n* = 6 for *t* = 1-1.5 h, *n* = 3 for other time points). *p* < 0.05 for LPS+PCERA-1 relative to LPS only at 1-2 h in the IL-10 assay (b, left) and at 1-3.5 h in the TNF*α* assay (b, right). Cytokine levels were undetected following administration of PCERA-1 alone or vehicle. The experiments were carried out twice.

The effect of isoproterenol on LPS-stimulated IL-10 secretion was verified in primary bone marrow-derived macrophages (BMDM). As for the RAW264.7 macrophage cell line, the high synergism between isoproterenol and LPS at 3 h was gradually diminished at later time points (Figure 2(a)). We suggest that the modest two-fold effect remaining for isoproterenol at 24 h reflects partial accumulation resulting from incomplete degradation/sequestration of IL-10 secreted from the BMDM at the early phase. Isoproterenol alone had no detectable activity (Figure 2(a)). To evaluate the physiological relevance of these findings *in vivo*, we measured serum IL-10 levels in mice injected with LPS and PCERA-1, a macrophage-specific cAMP-elevating agent that has been demonstrated by us to suppress TNF*α* expression and to synergistically drive IL-10 transcription via the cAMP pathway in LPS-stimulated primary and cultured macrophages, but not blood monocytes [12, 13, 16, 21].  Figure 2(b) (left panel) shows that LPS-induced serum IL-10 peaked earlier in PCERA-1-treated mice relative to control mice (1.5 h and 2.5 h, respectively) and that PCERA-1-elevated serum IL-10 levels within 2 h, but not later. In contrast, PCERA-1 continuously reduced LPS-induced serum TNF*α* levels (Figure 2(b), right panel). These findings thus suggest that the temporal regulation of IL-10 modulation in macrophages by cAMP-elevating agents is physiologically relevant in clinical contexts such as the mouse septic shock model.

### 3.2. Desensitization Does Not Account for the cAMP Insensitivity of Late LPS-Stimulated IL-10 Expression

**Figure 3:**
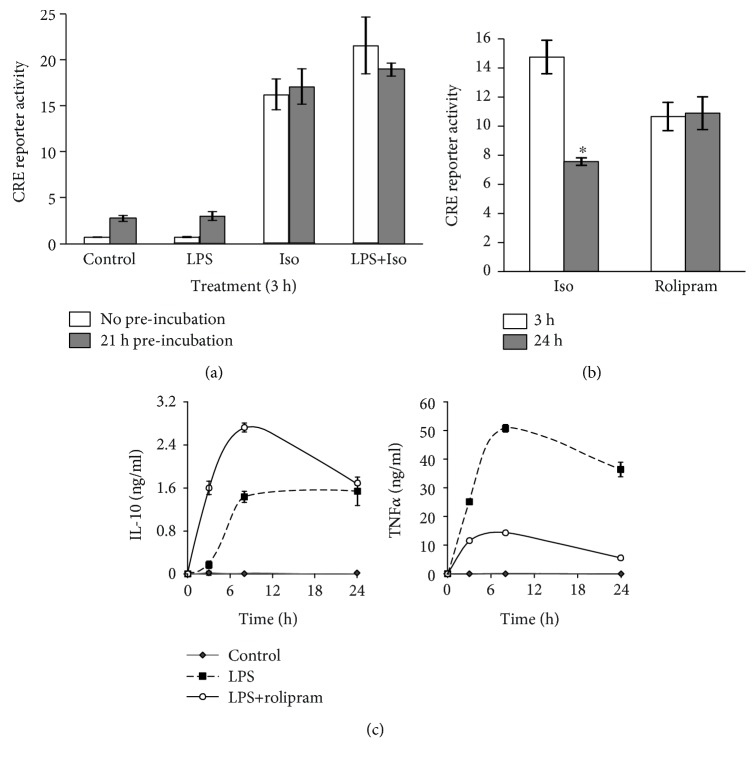
The insensitivity of late-phase IL-10 induction to isoproterenol does not result from partial receptor desensitization. (a, b) RAW264.7 macrophages were transfected with a luciferase reporter regulated by 4 repeats of a consensus CRE sequence. Luciferase reporter data expressed as the mean ± SD (*n* = 3) of values normalized against Renilla luciferase activity, relative to unstimulated control cells. (a) The cells were preincubated for 21 h with either LPS (10 ng/ml) or vehicle, washed and further incubated for the following 3 h with isoproterenol (Iso, 1 *μ*M) and/or LPS (10 ng/ml). (b) The cells were treated for 3 h or 24 h with isoproterenol (Iso, 1 *μ*M) or with the PDE4 inhibitor rolipram (20 *μ*M). ^∗^ *p* = 0.0002. (c) RAW264.7 macrophages were incubated for the indicated time with LPS (10 ng/ml) in the presence or absence of the PDE4 inhibitor rolipram (20 *μ*M). IL-10 and TNF*α* secretion was measured by ELISA. Data expressed as the mean ± SD (*n* = 6). *p* < 0.05 for LPS+rolipram relative to LPS alone at all time points except for IL-10 at 24 h. The experiments were carried out twice (a, c) or three times (b) with similar results.

The inability of isoproterenol to amplify LPS-stimulated IL-10 expression at the late phase could potentially be explained by cytotoxicity or by negative regulation of the cAMP pathway. Cytotoxicity was ruled out as neither LPS nor isoproterenol, alone or together, reduced cell viability at 24 h (Figure S1). To verify that a long LPS treatment does not impair CREB activity, we transfected RAW264.7 macrophages with a cAMP response element (CRE) consensus reporter, then preincubated the cells with LPS (or vehicle) for 21 h, followed by washing and further incubation for 3 h with isoproterenol.  Figure 3(a) shows that the capacity of isoproterenol to activate the CRE reporter was identical whether or not the cells were pretreated with LPS for 21 h and also whether or not the cells were cotreated (at the 3 h assay) with LPS. To examine the effect of *β*-AR desensitization, we again transfected RAW264.7 macrophages with a CRE consensus reporter and treated the cells with isoproterenol for 3 or 24 h. We found that the ability of isoproterenol to activate CRE-dependent transcription was reduced by only 50% in the 24 h assay, compared to the 3 h assay (Figure 3(b)). Moreover, to circumvent receptor desensitization, we employed rolipram, which elevates basal cAMP level by inhibiting its phosphodiesterase (PDE) 4-mediated degradation and found that although rolipram stimulated the CRE reporter to a similar extent at the early and late phases (Figure 3(b)), it synergized with LPS in IL-10 expression only at the early, but not late, phase (Figure 3(c), left panel). In contrast, the negative effect rolipram exerted on LPS-induced TNF*α* expression in the same cells was rather augmented along the experiment timecourse (Figure 3(c), right panel). Furthermore, rolipram mimicked isoproterenol in synergizing with LPS at IL-10 mRNA elevation only at the early phase, up to 4 h (Figure 1(f)). The effect of desensitization on isoproterenol's signaling was apparently echoed in the more rapid decline of IL-10 mRNA level, compared to rolipram (Figure 1(f)). Nevertheless, the synergistic effect of both compounds on LPS-stimulated IL-10 mRNA level was completely abolished at 8 h and later (Figure 1(f)). These results strongly argue against receptor desensitization as a major explanation for the abolished sensitivity of LPS-stimulated IL-10 induction to isoproterenol at the late phase, hinting to a switch in the mechanism of LPS itself with time.

### 3.3. The cAMP Pathway Does Not Cooperate with the LPS-Stimulated Autocrine Type I IFN Loop in IL-10 Expression at the Late Phase

**Figure 4:**
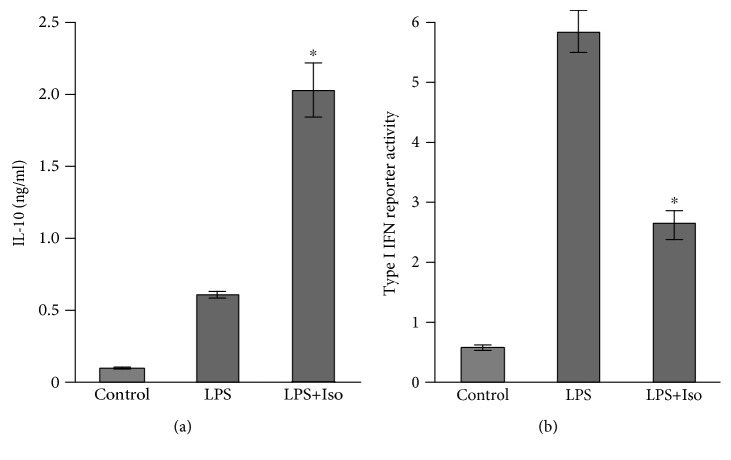
Isoproterenol inhibits LPS-stimulated type I IFN secretion. RAW264.7 macrophages were incubated with LPS (10 ng/ml)±isoproterenol (Iso, 1 *μ*M) for 1 h. The LPS-containing medium was then removed, and the conditioned medium was collected following 3 h without further stimulus. (a) IL-10 secretion from the macrophages was measured by ELISA. (b) Type I IFN level in the media was measured by a luciferase reporter ISRE-L929 cell line assay. Data expressed as the mean ± SD (*n* = 3) of values normalized against Renilla luciferase activity and divided by 10,000. ^∗^ *p* < 0.003 for cells costimulated with LPS+isoproterenol compared to cells stimulated with LPS alone. The experiment was carried out 5 times with similar results.

The temporal switch in the mechanism of LPS-stimulated IL-10 expression in macrophages may be related to the time-dependent indirect involvement of autocrine IFN [3–8]. We therefore initially asked whether the accelerating effect of isoproterenol on IL-10 expression in LPS-stimulated macrophages is mediated by an increase in the production of type I IFN at the early phase. To that end, we pretreated RAW264.7 macrophages for 1 h with LPS, alone or together with isoproterenol, removed the LPS-containing medium, collected the conditioned medium following the next 3 h of incubation in LPS-free medium, and analyzed the secretion of IL-10 by ELISA and of type I IFN by a luciferase reporter ISRE-L929 cell line [20]. We verified that the conditioned medium is indeed free of LPS by showing that the addition of the LPS antagonist polymyxin B to the conditioned medium had no effect on the signal (Figure S2). We found that while isoproterenol, as expected, increased early LPS-stimulated IL-10 secretion also under this protocol (Figure 4(a)), it negatively affected type I IFN activity in the medium of LPS-stimulated cells (Figure 4(b)). This finding indicates that the cAMP pathway does not amplify IL-10 expression indirectly via enhanced early LPS-stimulated autocrine type I IFN activity, and together with the data presented in Figure 1, it rather suggests that cAMP directly upregulates IL-10 promoter activity.

**Figure 5:**
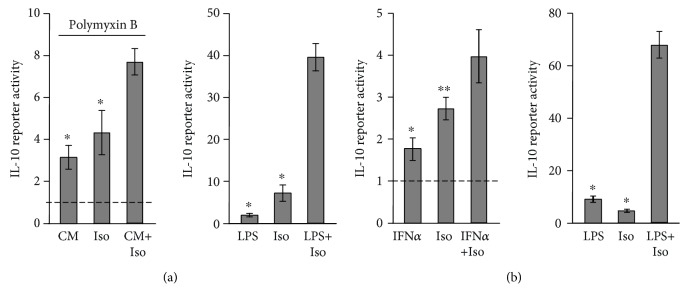
Isoproterenol synergistically amplifies only direct LPS-induced IL-10 transcription at the early phase, but not late, type I IFN-dependent LPS activity. (a, b) RAW264.7 macrophages were transfected with a full mouse IL-10 promoter reporter construct. (a) Left panel: nontransfected RAW264.7 macrophages were incubated for 21 h with either LPS (10 ng/ml) or vehicle, and the conditioned medium (CM) was collected. Then, the IL-10 promoter reporter cells were incubated for 3 h with either CM from LPS-treated cells in the presence or absence of isoproterenol (Iso, 1 *μ*M) or with CM from vehicle-treated cells together with isoproterenol. The LPS antagonist polymyxin B (50 *μ*M) was added to the CM in all treatments. Right panel: synergism between isoproterenol and LPS (10 ng/ml) is shown for comparison. (b) The IL-10 promoter reporter cells were incubated for 8 h with isoproterenol (Iso, 1 *μ*M) and/or either mouse IFN*α* (2000 units/ml, left panel) or LPS (10 ng/ml, right panel). (a, b) Luciferase reporter data expressed as the mean ± SD (*n* = 3) of values normalized against Renilla luciferase activity, relative to unstimulated control cells. ^∗^ *p* < 0.01, ^∗∗^ *p* < 0.03 for cells monostimulated relative to cells costimulated. The experiments were carried out twice with similar results.

As only late, but not early, IL-10 induction by LPS is type I IFN-dependent [3–8], we hypothesized that the cAMP pathway can amplify only the early-direct LPS activity at the IL-10 promoter, and not the late-indirect LPS activity, and set out to specifically measure indirect IL-10 promoter reporter activation at the late phase (24 h). To this end, RAW264.7 macrophages were transfected with the IL-10 promoter reporter and incubated for 3 h with isoproterenol and/or either of the following stimuli: LPS or conditioned medium (CM) collected from a different plate of RAW264.7 macrophages that were incubated with LPS alone or with vehicle for 21 h. Importantly, the LPS antagonist polymyxin B was added to the CM in order to block the activity of LPS present in the CM and so to enable cell stimulation only by autocrine/paracrine factors. Both LPS and the CM alone demonstrated low activity at the 3 h assay, but in contrast to the synergism isoproterenol displayed with LPS, it demonstrated only an additive effect with the CM in activating the IL-10 promoter reporter (Figure 5(a)), indicating that the cAMP pathway cannot synergize with the IL-10-inducing activity of autocrine factors secreted to the medium in response to a long (21 h) pretreatment with LPS. The efficiency of polymyxin B was verified in a separate experiment that showed 97% neutralization of LPS activity on the macrophages (Figure S2).

We then directly examined whether the cAMP pathway could amplify IL-10 induction by IFN*α*. Again, in contrast to the synergism displayed by LPS and isoproterenol, IFN*α* and isoproterenol demonstrated only an additive effect in IL-10 promoter reporter activation during the 8 h assay (Figure 5(b)), suggesting that late LPS-stimulated IL-10 expression is largely insensitive to cAMP-elevating agents because the autocrine type I IFN activity mediating this late indirect LPS effect does not synergize with the cAMP pathway. Notably, the low IL-10 promoter reporter induction by IFN*α*, relative to LPS (Figure 5(b)), suggests that the autocrine type I IFN loop is required, but not sufficient for maximal IL-10 expression in response to LPS.

### 3.4. MyD88 Is Required for Synergistic IL-10 Expression by LPS and cAMP

**Figure 6:**
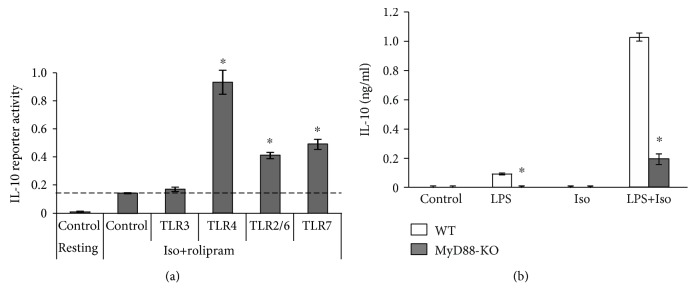
MyD88 is required for synergistic IL-10 expression by LPS and cAMP. (a) RAW264.7 macrophages were transfected with a full mouse IL-10 promoter reporter construct. The cells were treated for 3 h with a combination of isoproterenol (Iso, 1 *μ*M) and the PDE4 inhibitor rolipram (20 *μ*M), in the presence or absence of a TLR agonist, either LPS (10 ng/ml; TLR4), Pam2Cys (0.1 ng/ml; TLR2/6), poly(I:C) (50 *μ*g/ml; TLR3), or imiquimod (50 *μ*M; TLR7). Luciferase reporter data expressed as the mean ± SD (*n* = 3) of values normalized against Renilla luciferase activity. ^∗^ *p* < 0.0001 compared to control cells stimulated with isoproterenol+rolipram. Reporter activity following administration of any TLR agonist alone (without the cAMP inducers) was indistinguishable from control values. (b) BMDM from WT (C57BL/6) and corresponding MyD88 knockout mice were incubated for 3 h with LPS (10 ng/ml) and/or isoproterenol (Iso, 1 *μ*M). The medium was collected, and secretion of endogenous IL-10 was measured by ELISA. Data represent the mean ± SD (*n* = 6). ^∗^ *p* < 0.01 compared to WT. The experiments were carried out five (a) or three times (b) with similar results.

TLR4 signaling is mediated by two major adaptor proteins, MyD88 and TRIF [22]. In contrast, TLR2 heterodimers and TLR7 signal only via MyD88, whereas TLR3 signals exclusively via TRIF [22]. We therefore asked which adaptor protein mediates the synergism between LPS and the cAMP pathway at the early phase of IL-10 expression. To begin answering this question, we compared the ability of cAMP to synergize with agonists of the different TLRs: LPS (TLR4), Pam2Cys (TLR2/6), poly(I:C) (TLR3), and imiquimod (TLR7). In this experiment, in order to maximally elevate cAMP, we used a combination of the *β*-AR agonist isoproterenol and the PDE4 inhibitor rolipram. The LPS concentration used throughout this study was 10 ng/ml, which is equal to the EC_50_ for IL-10 induction ([12] and Figure S3). Due to their lower activity, Pam2Cys and imiquimod were used at concentrations which are 1.5-fold their EC_50_ (Figure S3), and poly(I:C) was used at a concentration that ensures maximal activity [23]. Importantly, we have previously shown that the synergistic effect of isoproterenol on LPS-induced IL-10 expression was similar at the LPS concentration range of 10-1000 ng/ml [12]. Likewise, the synergistic effect of isoproterenol on imiquimod-induced IL-10 expression was similar at the imiquimod concentration range of 10-100 *μ*M [12]. We found that synergistic IL-10 promoter reporter activation exists only between the cAMP inducers and agonists of MyD88-coupled TLRs, LPS, Pam2Cys, and imiquimod, but not with poly(I:C) which signals independently of MyD88 (Figure 6(a)). As a positive control for poly(I:C) functionality in the macrophages, we determined that while poly(I:C) was unable to significantly induce IL-10 promoter reporter activity, it dramatically induced C-X-C motif chemokine 10 (CXCL10) (Figure S4).

To verify that indeed MyD88 mediates the synergism between LPS-activated TLR4 and the cAMP pathway, we isolated BMDM from WT and MyD88 knockout mice and incubated them with LPS and/or isoproterenol for 3 h.  Figure 6(b) shows that isoproterenol amplified the low LPS-induced IL-10 secretion by 11-fold in WT macrophages, while MyD88 knockout abolished 80% of the synergistic IL-10 expression. Our results therefore indicate that MyD88 plays a major role in the ability of LPS-activated TLR4 to induce IL-10 at the early phase, alone and synergistically with the cAMP pathway.

## 4. Discussion

Stimulation of macrophages by LPS drives massive production of proinflammatory cytokines such as TNF-*α* and IL-12 and only low levels of IL-10. The subsequent anti-inflammatory response of the immune system involves a macrophage phenotype characterized by secretion of higher levels of IL-10 and lower levels of proinflammatory cytokines. Such a phenotype can also be obtained by the combination of a TLR ligand (e.g., LPS) and a second stimulus (e.g., IgG immune complex, apoptotic cell remnants, or a cAMP inducer) that reprograms the LPS-stimulated macrophage to become anti-inflammatory [1, 2].

Our lab previously reported that various cAMP-elevating agents (i.e., isoproterenol, PGE_2_, and the synthetic phospholipid PCERA-1) induce IL-10 in mouse macrophages via PKA in synergism with LPS [12]. We therefore sought to explore the time dependency of this synergism. We found that cAMP imposes temporal regulation on IL-10 transcription in LPS-stimulated macrophages, by synergizing with LPS only at the early, but not late, phase. This was demonstrated on the levels of IL-10 promoter reporter, mRNA expression, and protein secretion. Furthermore, this finding was recapitulated in primary macrophages and also *in vivo* in a LPS-induced mouse septic shock model using PCERA-1, a synthetic anti-inflammatory compound which dramatically increases mice survival [17], apparently by elevating IL-10 and suppressing TNF*α* expression in macrophages via the cAMP pathway [12, 13, 16, 21]. *In vivo*, the cAMP-elevating agent increased serum IL-10 levels only at the early stage (up to 2 h), but not at later time points, in contrast to its persistent ability to suppress serum TNF*α* levels throughout the entire time range.

LPS has a more profound effect on early IL-10 secretion than on IL-10 promoter reporter induction, pointing to a posttranscriptional mechanism for IL-10 induction by LPS alone at the early phase. Accordingly, LPS significantly increased early IL-10 mRNA level, consistent with a previous report by Pattison et al. [8]. In line with this, the 3′-UTR of the mouse IL-10 mRNA mediates transcript decay, which is reversed by LPS-stimulated p38 [24, 25]. As shown here, the cAMP pathway plays a permissive role in IL-10 secretion as it can modestly increase transcription rate but is unable to stimulate the posttranscriptional step that is limiting secretion. Therefore, accelerated IL-10 expression in cells costimulated by LPS and a cAMP-elevating agent apparently results from synergistic transcription combined with LPS-dependent increase in mRNA stability.

We show that the adaptor protein MyD88 is required for the synergistic IL-10 induction at the early phase in macrophages costimulated by a cAMP-elevating agent and the TLR4 agonist LPS. Consistently, the cAMP pathway synergistically induces the IL-10 promoter reporter also with agonists of TLR2/6 and TLR7, which couple to MyD88, but not with an agonist of TLR3 which signals independently of MyD88. Yet we cannot exclude the possibility that the TRIF pathway contributes to the synergism between TLR4 and the cAMP pathway regarding IL-10 induction. Such a scenario may explain the inability of MyD88 knockout to completely abolish synergistic IL-10 expression in macrophages costimulated with LPS and isoproterenol.

The inability of cAMP-elevating agents to amplify late LPS-stimulated expression is not due to receptor desensitization which partially reduces cAMP signaling, as we show that isoproterenol does activate a CRE consensus reporter even at 24 h and, importantly, that the PDE4 inhibitor rolipram is also able to affect only early IL-10 expression. These results suggest that LPS-stimulated IL-10 transcription is carried out by different mechanisms at the early and late phases.

LPS-stimulated IL-10 mRNA expression requires, at least in part, de novo protein translation [15, 26]. Indeed, IL-10 expression by LPS lags behind and is largely dependent on the expression and autocrine activity of type I IFNs [3–6, 8], which in turn stimulate the expression and autocrine IL-10 induction activity of IL-27 [7]. The second objective set in this study was to examine the possibility of crosstalk affecting IL-10 expression between the cAMP pathway and the autocrine type I IFN loop. Interestingly, we found opposite time dependencies for IL-10 modulation by these pathways. In contrast to the synergism that cAMP-elevating agents display with early type I IFN-independent IL-10 expression by LPS, cAMP is unable to amplify the late type I IFN-dependent activity. The latter is apparently due to the inability of cAMP to synergize with exogenous IFN*α* or with autocrine factors secreted to the conditioned medium during a long LPS stimulation. Our findings suggest that signaling downstream to cAMP and LPS cooperatively drive IL-10 expression at the early phase, but not later when LPS-stimulated macrophages switch to type I IFN-dependent IL-10 expression. Consistently, the cAMP-elevating neuropeptide CGRP is able to enhance only early type I IFN-independent IL-10 expression in LPS-stimulated macrophages [3]. Furthermore, we found that LPS-stimulated secretion of type I IFN is reduced by the cAMP pathway, possibly due to the increased IL-10 secretion that may negatively regulate IFN*β* expression in an autocrine manner [27]. Alternatively, this may be a primary cAMP effect mediated by EPAC, as reported for the inhibition of IFN*β* expression by the cAMP-elevating agent PGE_2_ in J774A.1 macrophages [28].

Our results suggest that there are distinct time-dependent activities for cAMP and type I IFN, regarding IL-10 induction in LPS-stimulated cells. Specifically, enhanced IL-10 expression by cAMP-elevating agents occurs only at the early phase, while LPS-stimulated type I IFNs are involved in IL-10 expression at the late phase. As this was demonstrated in an IL-10 promoter reporter assay, it is likely due to discrete regulation of IL-10 transcription. Type I IFNs were reported to stimulate IL-10 expression via multiple transcription factor binding sites at the IL-10 promoter, including Sp1, STAT1/3, and c-Maf [7]. Elevation of cAMP stimulates IL-10 expression in human cells via several CRE sequences, one of which is conserved also in the mouse promoter [29] that was examined in this study. We suggest that transcription factors that are stimulated by LPS only at the late phase, in part via the autocrine type I IFN loop, activate the IL-10 promoter to the extent that the cAMP-dependent transcription factor (e.g., CREB) cannot further increase the IL-10 transcription rate.

Opportunistic Gram-negative bacteria, such as *Pseudomonas aeruginosa*, secrete a quorum sensing molecule named N-3-oxo-dodecanoyl-L-homoserine lactone (hereafter, C12) which directly modulates vital bacterial as well as host functions, thereby promoting establishment of chronic infections [30, 31]. C12 reduces inflammation by suppressing expression of the key proinflammatory cytokine TNF*α* in LPS-stimulated macrophages [32]. In contrast, we have previously reported that C12 synergistically amplifies LPS-stimulated IL-10 expression in RAW264.7 and peritoneal macrophages [33]. Thus, it is suggested that C12 promotes chronic bacterial infection in part by downregulating inflammation through its reciprocal effects on the expression of pro- and anti-inflammatory cytokines. As yet, neither the mammalian receptor nor the signaling mechanism for cytokine modulation is known. Interestingly, in epithelial cells, some C12 effects are mediated by a decrease in calcium concentration in the ER and a subsequent store-operated cAMP production [34]. Thus, if C12 can similarly elicit cAMP production in macrophages that may provide a mechanism for the reciprocal IL-10 and TNF*α* modulation by C12 in LPS-stimulated cells, in line with the evidence, we presented here and previously [14] the respective positive vs. negative roles of cAMP in expression of these cytokines.

## 5. Conclusions

The slow induction of IL-10 by LPS allows a proper innate immune response to pathogens. The lag in IL-10 secretion from macrophages, relative to proinflammatory cytokines, is explained by the inability of LPS to directly stimulate early IL-10 transcription in a significant manner and its dependence on the late indirect autocrine activity of LPS-induced type I IFN. A costimulus elevating macrophage intracellular cAMP level overcomes the requirement for the late autocrine type I IFN loop and accelerates IL-10 induction by synergizing in a MyD88-dependent manner with the minor direct effect of LPS on early IL-10 transcription and with its more prominent posttranscriptional regulation. By doing so, the cAMP pathway promotes an anti-inflammatory feature on macrophages, to dampen the innate immune response. In a clinical setting, administration of macrophages characterized by increased IL-10 secretion attenuates the development of acute sepsis and chronic autoimmune diseases such as colitis [9]. Aberrant endogenous IL-10 expression may be physiologically relevant for example under stress, a condition characterized by secretion of multiple primary messengers which activate receptors (expressed in macrophages) upstream of the cAMP pathway, including adrenaline [35], CRH [36], *α*-MSH, and ACTH [37]. In line with this, stress upregulates *in vivo* levels of IL-10 leading to immune depression [38]. Therefore, stimulation of G_s_-coupled receptors (upstream to cAMP production) which are expressed in macrophages may provide therapeutic means to limit exaggerated inflammatory responses and by that to reduce organ damage and mortality, as demonstrated in murine septic shock models [39].

## Data Availability

The data used to support the findings of this study are included within the article and the supplementary information file.

## Abbreviations

*β*-AR: *β*-Adrenergic receptor
BMDM: Bone marrow-derived macrophages
CM: Conditioned medium
CRE: cAMP response element
CREB: cAMP response element binding protein
CXCL10: C-X-C motif chemokine 10
IBD: Inflammatory bowel disease
IFN(s): Interferon(s)
IL-10: Interleukin-10
Iso: Isoproterenol
LPS: Lipopolysaccharide
PCERA-1: Phospho-ceramide analogue-1
PDE: Phosphodiesterase
TLR: Toll-like receptor
TNF*α*: Tumor necrosis factor alpha.

## Supplementary Materials

Supplementary Materials Figure S1. Cell viability. Figure S2. Polymyxin B abolishes LPS activity. Figure S3. Dose responses for TLR agonists. Figure S4. CXCL10 induction by poly(I:C).
