# Supplementary material for: The cAMP Pathway Amplifies Early MyD88-Dependent and Type I Interferon-Independent LPS-Induced Interleukin-10 Expression in Mouse Macrophages
Source: Mediators Inflamm. 2019 Apr 17;2019:3451461. doi: 10.1155/2019/3451461 (PMC6501241; doi:10.1155/2019/3451461)
Supplement: Supplementary Materials — Figure S1. Cell viability. Figure S2. Polymyxin B abolishes LPS activity. Figure S3. Dose responses for TLR agonists. Figure S4. CXCL10 induction by poly(I:C). [file 3451461.f1.doc]

**The cAMP pathway amplifies early MyD88-dependent and type I interferon-independent LPS-induced interleukin-10 expression in mouse macrophages**

**Orna Ernst**^1,2^**, Yifat Glucksam-Galnoy**^1^**, Muhammad Athamna**^1,3^**, Iris Ben-Dror**^1^**, Hadar Ben- Arosh**^1^**, Galit Levy-Rimler**^1^**, Iain D.C. Fraser**^2^ **and Tsaffrir Zor**^1^

**Supplementary Information**

**
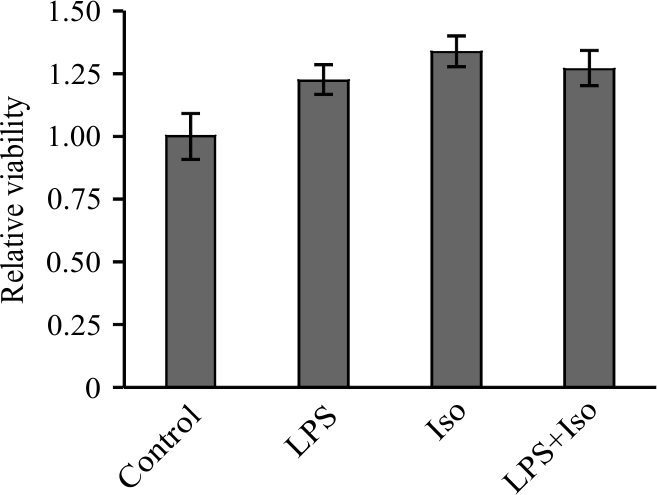
**

**Fig. S1.** Stimulation of RAW264.7 macrophages with LPS and/or isoproterenol does not impair viability. RAW264.7 macrophages were incubated with LPS (10 ng/ml) and/or isoproterenol (Iso, 1 µM) for 24 h. Viability was determined by an XTT assay. Data represent mean ± SD (n=6).

**
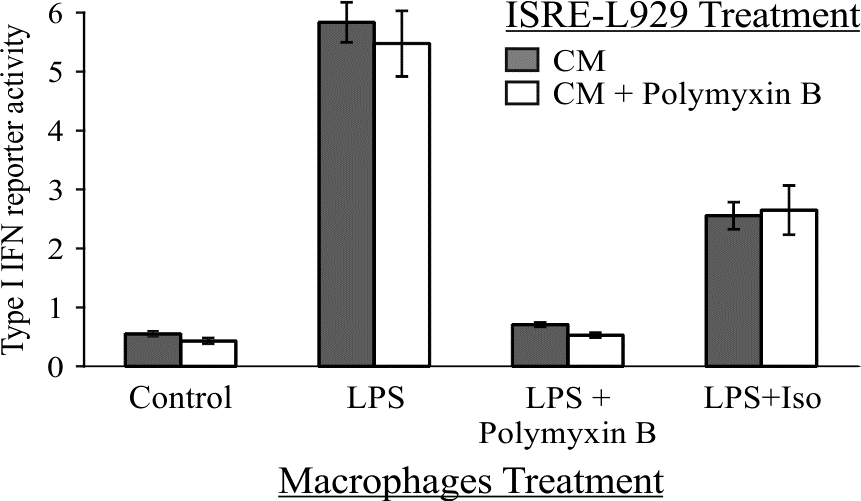
**

**Fig. S2.** Polymyxin B blocks 97% of LPS activity. RAW264.7 macrophages were incubated for 1 h with LPS (10 ng/ml) with or without isoproterenol (Iso, 1 μM) or the LPS antagonist polymyxin B (50 µM). The LPS-containing medium was then removed and the conditioned medium was collected following 3 h without further stimulus. Type I IFN level in the media was measured by a luciferase reporter ISRE-L929 cell line assay. Open bars represent type I IFN activity in conditioned media (CM) to which polymyxin B (50 µM) was added. There is no statistically significant difference between open and solid bars, indicating that indeed the CM is LPS-free. Data expressed as mean ± SD (n=3) of values normalized against renilla luciferase activity and divided by 10,000. The solid bars of control, LPS and LPS + isoproterenol are shown also in Fig. 4B. The abolished activity of macrophages treated by LPS together with polymyxin B indicates that polymyxin B fully chelates LPS.


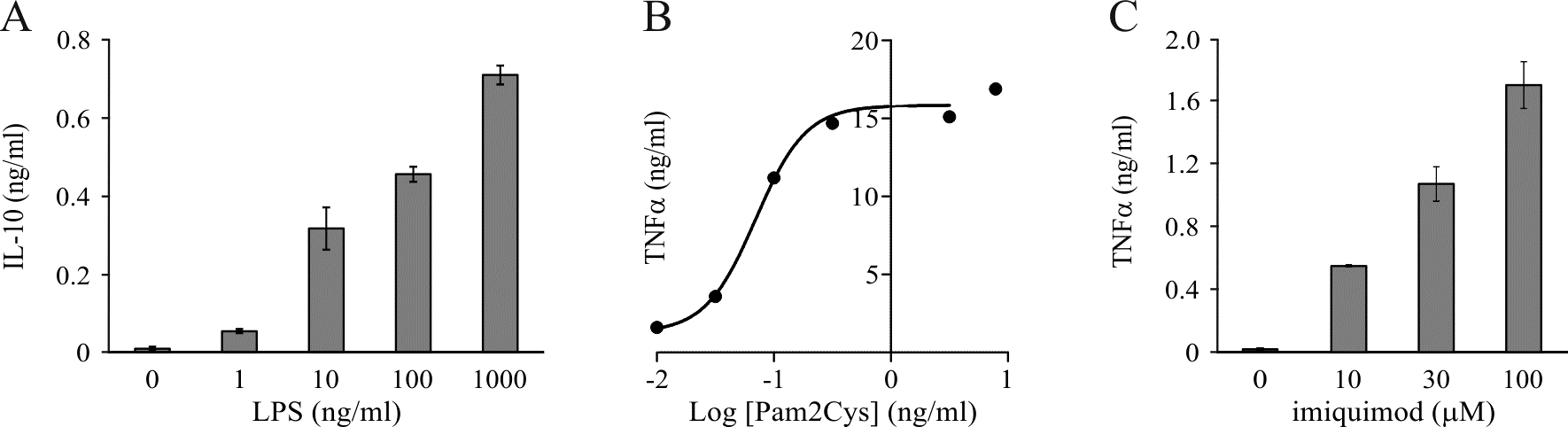


**Fig. S3.** Dose responses for TLR agonists. RAW264.7 macrophages were incubated with the indicated concentrations of LPS, Pam2Cys or imiquimod for 4 h (**A**), 24 h (**B**) or 2 h (**C**). IL-10 and TNFα secretion were measured by ELISA. Data expressed as mean ± SD (n=6). The estimated EC_50_ values for LPS and imiquimod are 10 ng/ml and 30 μM, respectively. The EC_50_ for Pam2Cys was calculated (using the software GraphPad Prism) to be 0.07 ng/ml.

**
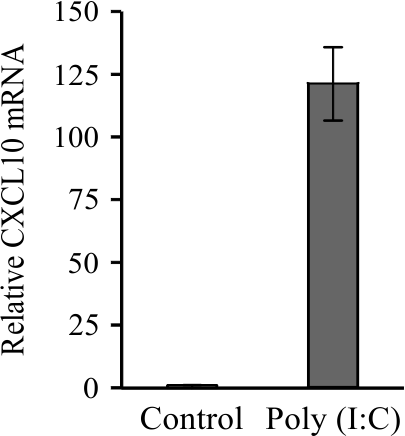
**

**Fig. S4.** Poly (I:C) induces the chemokine CXCL10. RAW264.7 macrophages were incubated with the TLR3 agonist Poly (I:C) (20 µg/ml) for 4 h. Total RNA was isolated from the cells, and CXCL10 mRNA levels were assessed by real time PCR. The intensity of CXCL10 mRNA in unstimulated cells, normalized by HPRT mRNA, was set to 1. Data represent mean ± SD (n=4).
